# Neuroprotective effect of astragalin via activating PI3K/Akt-mTOR-mediated autophagy on APP/PS1 mice

**DOI:** 10.1038/s41420-023-01324-1

**Published:** 2023-01-21

**Authors:** Cui-Zhu Yang, Shu-Han Wang, Run-Heng Zhang, Jia-Hong Lin, Ying-Hong Tian, Ya-Qi Yang, Jing Liu, Yu-Xin Ma

**Affiliations:** 1 Department of Anatomy, School of Life Sciences and Biopharmaceutics, Guangdong Pharmaceutical University, Guangzhou, China; 2 Experiment Teaching & Administration Center, School of Basic Medical Sciences, Southern Medical University, Guangzhou, China; 3 Guangdong Key Laboratory of Pharmaceutical Bioactive Substances, Guangdong Pharmaceutical University, Guangzhou, China

**Keywords:** Alzheimer's disease, Hippocampus

## Abstract

As a small molecule flavonoid, astragalin (AST) has anti-inflammatory, anti-cancer, and anti-oxidation effects. However, the impact and molecular mechanism of AST in Alzheimer’s disease (AD) are still not clear. This study aims to investigate the neuroprotective effect and mechanism of AST on APP/PS1 mice and Aβ25-35-injured HT22 cells. In this study, we found that AST ameliorated cognitive dysfunction, reduced hippocampal neuronal damage and loss, and Aβ pathology in APP/PS1 mice. Subsequently, AST activated autophagy and up-regulated the levels of autophagic flux-related protein in APP/PS1 mice and Aβ25-35-induced injury in HT22 cells. Interestingly, AST down-regulated the phosphorylation level of PI3K/Akt-mTOR pathway-related proteins, which was reversed by autophagy inhibitors 3-Methyladenine (3-MA) or Bafilomycin A1 (Baf A1). At the same time, consistent with the impacts of Akt inhibitor MK2206 and mTOR inhibitor rapamycin, inhibited levels of autophagy in Aβ25-35-injured HT22 cells were activated by the administration of AST. Taken together, these results suggested that AST played key neuroprotective roles on AD via stimulating PI3K/Akt-mTOR pathway-mediated autophagy and autophagic flux. This study revealed a new mechanism of autophagy regulation behind the neuroprotection impact of AST for AD treatment.

## Introduction

Alzheimer’s disease (AD), the most common age-related neurodegenerative disease, accounts for 50–70% of all dementia patients and is typically characterized by progressive cognitive decline [1]. With the continuous improvement of quality of life and medical technology, the global elderly population is showing a growing trend. AD has caused more deaths among patients than the total number of breast and prostate cancer, and the number of AD patients aged 65 and above is expected to reach 7.2 million by 2025 [2]. The pathogenesis of AD is extremely complicated, and research on its pathogenesis has focused on abnormal accumulation of extracellular amyloid-β (Aβ), intracellular tau protein hyperphosphorylation, synaptic loss and neuronal death, and cholinergic nerve damage. At present, drugs developed for the above pathogenesis of AD are not well applied in the clinical, such as soluble Aβ protein specific binding agent Solanezumab [3], targeted inhibition of tau protein RO7105705 and LY3303560 [4], and cholinesterase inhibitors galantamine [5], etc. Therefore, the development of novel and effective natural drugs to prevent and improve AD pathology is in particular indispensable.

Autophagy, as a degradation mechanism, can remove intracellular injured and aging organelles and is imperative for maintaining intracellular homeostasis [6]. Early in the onset of AD patients, activation of autophagy serves as a protective mechanism to reduce neuronal cell damage. And with the progression of AD, autophagy is restrained. The accumulated Aβ in the brain of AD patients prohibits the degradation of lysosomes, further blocking the digestion and degradation pathway of autophagic lysosomes to the contents and thus causing cell death [7, 8]. Therefore, dysfunction of autophagy may be an essential pathological mechanism leading to the accumulation of intracellular Aβ peptides, further causing neuronal damage and the formation of senile plaques (SPs) deposited in the AD brain. Autophagic flux is the whole procedure of autophagy, including autophagy initiation, autophagosome formation, autolysosome fusion and degradation [9]. And anomalies in any of these steps can cause excessive aggregation of SPs in the brain of AD patients [10]. Therefore, reversing autophagy or autophagic flux dysfunction may be an innovative therapeutic target for AD.

Astragalin (AST) is a natural flavonoid with anti-inflammatory [11], anti-cancer [12], and neuroprotective [13], effects. Available studies have reported that AST could protect against cerebral ischemia-reperfusion injury by weakening oxidative stress, inflammation response, and apoptosis [14]. It also has been reported that AST diminished the production of TNF-α, IL-6, and IL-1β by inactivating the NF-κB pathway in a mouse model of LPS-induced acute lung injury [15]. However, whether AST can exert a neuroprotective role in neurological diseases such as AD by regulating autophagy or autophagic flux has not been reported. Therefore, in this study, we aimed to investigate the impact and molecular mechanism of AST on cognitive dysfunction in APP/PS1 mice and to provide basic theoretical support for further revealing the potential role of AST in AD treatment.

## Results

### AST exists in numerous Chinese herbal medicines for the treatment of AD

First, in order to better select the appropriate drug, we used the TCMSP database to set the screening condition as drug-like property (DL) ≥ 0.18, which yielded that AST existed in many traditional Chinese medicines in the AD treatment, such as Acori tataninowii Rhizoma, Eucommiae Cortex, Paeoniae Radix Alba, Carthami Flos, Forsythiae Fructus, Achyranthis Bidentatae Radix, Granati Pericarpium, and Epimrdii Herba (Table 1).

**Table 1:** Part of the components of Chinese herbal medicines for the treatment of AD.

Traditional Chinese medicine	Partial molecule name	Mechanism of action	Reference
Acori tataninowii Rhizoma	8-Isopentenyl-kaempferol, Cycloartenol, beta-asarone, Astragalin	promoted the growth of neurons as well as the growth and connection of neuronal synapses	[51]
Eucommiae Cortex	protocatechuic acid, vanillic acid, Trochol, Astragalin	involved in the activation of the cholinergic system via inhibition AChE and TBARS activities	[52]
Paeoniae Radix Alba	propyl (2 R)-2-hydroxypropanoate, gallotannin, paeonoside, Astragalin	prevented oxidative injury and mitochondrial dysfunction	[53]
Carthami Flos	o-xylene, p-xylene, Arachic acid, Astragalin	inhibited oxidative stress damage and cell apoptosis	[54]
Forsythiae Fructus	vanillic acid, Cymol, citral, Astragalin	inhibited NF-κB signaling pathway	[55]
Achyranthis Bidentatae Radix	poriferasta-7,22E-dien-3beta-ol, 3-epioleanolic acid, Astragalin	reduced the accumulation of advanced glycation end products	[56]
Granati Pericarpium	Officinalisin, Punicalin, cyclohexane, Astragalin	inhibited nuclear factor of activated T-cell activity and microglial activation	[57]
Epimrdii Herba	(L)-alpha-Terpineol, isoliquiritigenin, Astragalin	promoted neuronal cell activity, preserved mitochondrial and synaptic functional proteins	[58]

### AST treatment ameliorates cognitive dysfunction of APP/PS1 mice

**Fig. 1:**
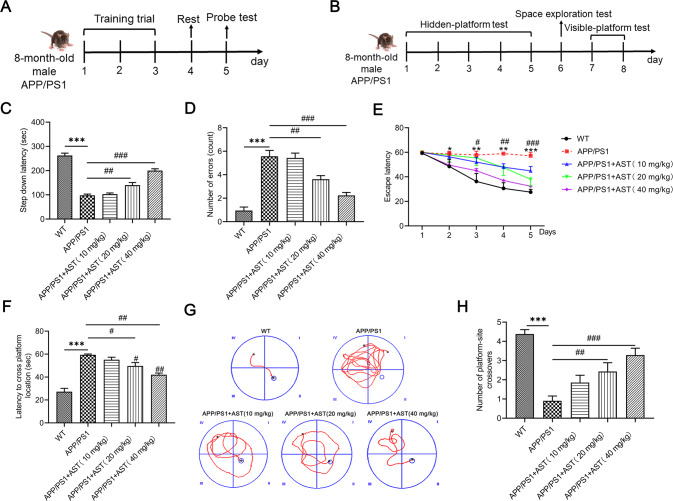
AST improved the cognitive dysfunction of APP/PS1 mice in the step-down avoidance (SDA) test and the Morris water maze (MWM) test. **A**, **B** Timelines of the mice SDA and MWM test. **C**, **D** The step-down latency and the number of errors of mice in the SDA test. **E** The escape latency of mice to arrive at the platform during the positioning navigation test in the MWM test (Day 1–5). **F**, **G** Time of mice to reach the target platform and swimming trajectory diagrams of mice during the space exploration in the MWM test (Day 6). **H** The number of mice crossing the target platform during the visible platform period in the MWM test (Day 7–8). Data were presented as mean ± SD, *n* = 7/group. *p < 0.05, **p < 0.01 and ***p < 0.001 vs WT group, ^#^*p* < 0.05, ^##^*p* < 0.01 and ^###^*p* < 0.001 vs APP/PS1 group.

To explore the impact of AST on the cognitive dysfunction of APP/PS1 mice, we performed the Step-down Passive Avoidance (SDA) test and Morris Water Maze (MWM) test (Fig. 1A, B). We observed that in the SDA test, compared with C57BL/6 (WT) mice, the step-down latency was shortened and the number of errors was increased in APP/PS1 mice, while these changes were inverted by administration of 20, 40 mg/kg AST (Fig. 1C, D). In the MWM test, APP/PS1 mice showed longer escape latency than WT mice during the training period, whereas AST significantly shorten the escape latency of APP/PS1 mice (Fig. 1E). During the trial period, APP/PS1 mice had significantly longer time to find the target quadrant and significantly lower frequency to reach the target platform when compared with the WT mice and the mice by administration of AST (Fig. 1F–H). Results above suggested that AST improved the cognitive deficits of APP/PS1 mice.

### AST attenuates hippocampal neuronal damage of APP/PS1 mice

**Fig. 2:**
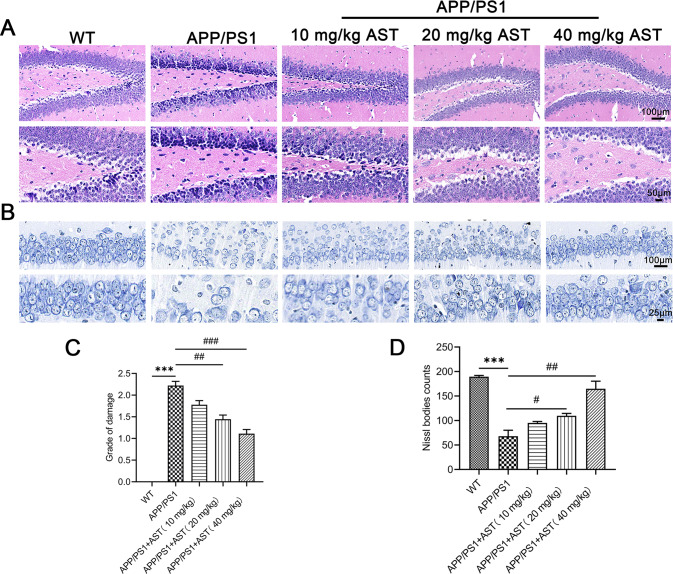
AST reduced hippocampal neuronal damage of APP/PS1 mice by HE and Nissl staining. **A** HE staining aimed to observe the alterations of neuronal morphology in mice hippocampus. Scale bar = 100, 50 μm, respectively. **B** Nissl staining aimed to detect changes in the morphology of Nissl bodies in the mice hippocampus. Scale bar = 100, 25 μm, respectively. **C** The grade of neuronal damage in the hippocampus of mice. **D** Statistical analysis of the Nissl bodies in the hippocampus of mice. Data were presented as mean ± SD, *n* = 4/group. ***p < 0.001 vs WT group, ^#^*p* < 0.05 and ^##^*p* < 0.01 vs APP/PS1 group.

The spatial learning and memory abilities are closely related to hippocampal structures. Therefore, we further examined the impact of AST on neuron damage in the hippocampus of APP/PS1 mice by HE and Nissl staining. The obvious neuronal damage was found in the hippocampus of APP/PS1 mice, and the neuronal damage grade score was increased in contrast with WT mice (Fig. 2A, C). While AST treatment significantly attenuated neuronal damage, the score of neuronal damage grade was significantly lower in the hippocampus of animals (Fig. 2A, C). Additionally, the number of Nissl bodies in the hippocampus of APP/PS1 mice decreased when compared to WT mice (Fig. 2B, D), whereas the number of Nissl bodies in the hippocampus of APP/PS1 mice increased by administration of AST 20, 40 mg/kg AST (Fig. 2B, D). The HE and Nissl staining results suggested that AST alleviated the histopathological changes and neuronal loss in the hippocampus of APP/PS1 mice.

### AST treatment reduces Aβ accumulation in the brain of APP/PS1 mice

**Fig. 3:**
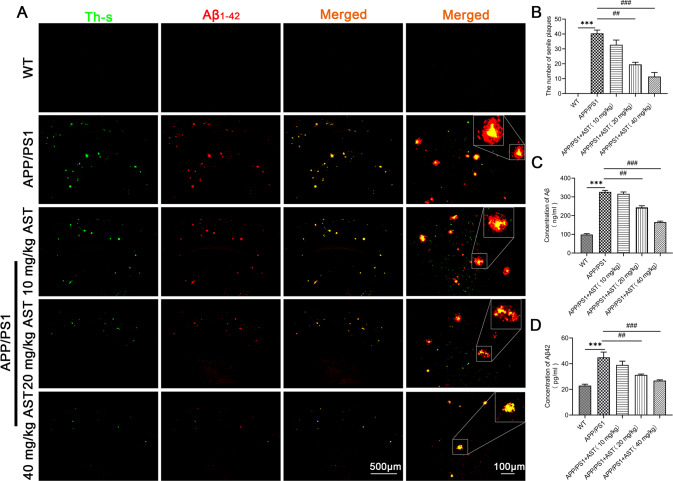
AST reduced Aβ aggregation in the APP/PS1 mice. **A** Detection of SPs in the brain of mice by Th-S staining and immunofluorescent co-staining. Scale bar = 500 μm in the three columns of images, and scale bar = 100 μm in the last column of images. **B** Statistical analysis of the number of SPs in the brain of mice in the merged images at 5X magnification (scale bar = 500 μm) of **A**. **C**, **D** The Aβ and Aβ42 levels in the serum of mice were detected by ELISA. Data were presented as mean ± SD, *n* = 3/group. ***p < 0.001 vs WT group, ^##^*p* < 0.01 and ^###^*p* < 0.001 vs APP/PS1 group.

The production and accumulation of large amounts of Aβ peptides can promote the formation of SPs and lead to cognitive impairment in APP/PS1 mice. Thus, we subsequently detected Aβ1-42 peptides in the brain of APP/PS1 mice by administration of AST. The quantitative analysis indicated there were many SPs in the brain of APP/PS1 mice (Fig. 3A, B). whereas with the administration of AST, SPs were considerably diminished in a dose-dependent manner, especially by administration of 40 mg/kg AST (Fig. 3A, B). Additionally, we also estimated the effect of AST on soluble and insoluble Aβ and Aβ42 in the serum of all mice. Compared with WT mice, the levels of Aβ and Aβ42 in the serum of APP/PS1 mice were higher, while Aβ and Aβ42 levels in the serum of APP/PS1 mice were decreased by administration of 20 and 40 mg/kg AST (Fig. 3C, D). The results indicated that AST may reduce the level of SPs in the brain of APP/PS1 mice.

### AST treatment activates hippocampal neuronal autophagy in APP/PS1 mice

**Fig. 4:**
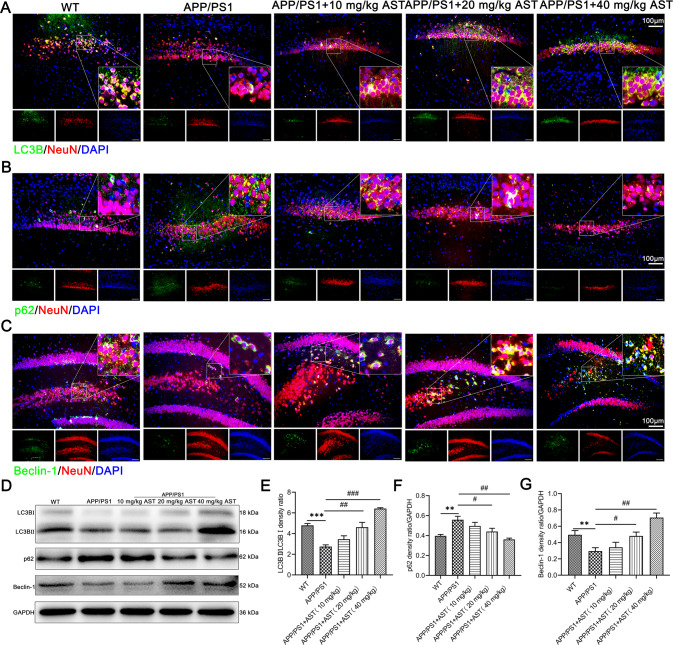
AST activated autophagy initiation in hippocampal neurons of APP/PS1 mice. **A**–**C** Representative immunofluorescent staining of LC3B/p62/Beclin-1, NeuN, and DAPI in hippocampal neurons of mice. Scale bar = 100 μm. **D** Representative bands of LC3BII/LC3BI, p62, and Beclin-1 in the hippocampus of mice by western blot (WB) detection. **E**–**G** Statistical analysis of LC3BII/LC3BI, p62, and Beclin-1 proteins in the hippocampus of mice. Data were presented as mean ± SD, *n* = 3/group. **p < 0.01 and ***p < 0.001 vs WT group, ^#^*p* < 0.05, ^##^*p* < 0.01 and ^###^*p* < 0.001 vs APP/PS1 group.

To study the effect of AST on autophagy function, we observed the expression of LC3B, p62, and Beclin-1 involved in the initiation of autophagy by administration of AST. Interestingly, we observed LC3B was mainly located in the cytoplasm of hippocampal neurons of mice (Fig. 4A), p62 was mainly located in the cytoplasm and nucleus of hippocampal neurons of mice (Fig. 4B), and Beclin-1 was mainly located in the cytoplasm and membrane of hippocampal neurons of mice (Fig. 4C). The ratio of LC3BII/LC3BI and the expression of Beclin-1 in APP/PS1 mice hippocampus were lower than those in WT mice (Fig. 4D, E, G), while the expression of p62 was higher (Fig. 4D, F). Notably, AST enhanced the ratio of LC3BII/LC3BI and the expression of Beclin-1 (Fig. 4D, E, G), but decreased the expression of p62 (Fig. 4D, F). The data suggested AST activated the autophagy of hippocampal neurons in APP/PS1 mice.

### AST treatment promotes autophagosome formation and autophagolysosome degradation in hippocampal neurons of APP/PS1 mice

**Fig. 5:**
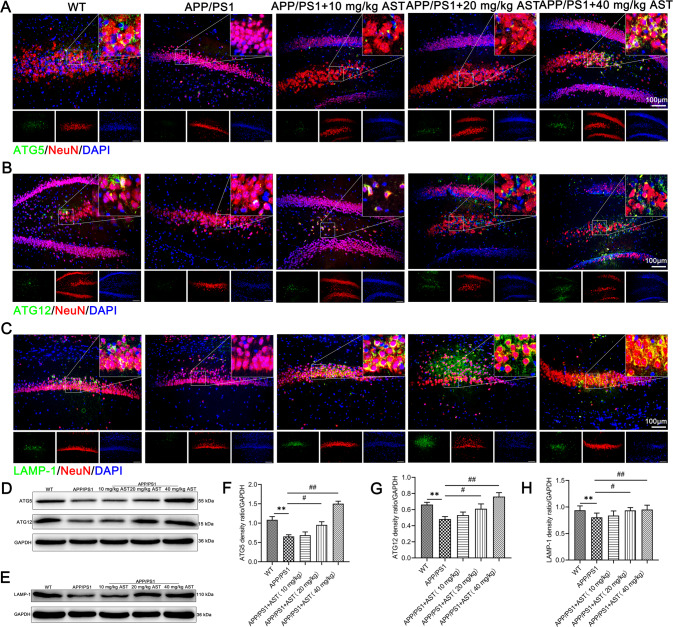
AST promoted autophagosome formation and the initiation of autophagic lysosomal phase in hippocampal neurons of APP/PS1 mice. **A**–**C** Representative immunofluorescent staining of ATG5/ATG12/LAMP-1, NeuN, and DAPI in hippocampal neurons of mice. Scale bar = 100 μm. **D**, **E** Representative bands of ATG5, ATG12, and LAMP-1 in the hippocampus of mice by WB detection. **F**–**H** Statistical analysis of ATG5, ATG12, and LAMP-1 proteins in the hippocampus of mice. Data were presented as mean ± SD, *n* = 3/group. **p < 0.01 vs WT group, ^#^*p* < 0.05, ^##^*p* < 0.01 vs APP/PS1 group.

Next, we detected the expressions of ATG5, ATG12, and LAMP-1 involved in autophagosome formation and autophagosome degradation in hippocampal neurons of mice. We observed ATG5 and ATG12 were mainly located in the cytoplasm of hippocampal neurons of mice (Fig. 5A, B) and LAMP-1 was mainly located in the membrane of hippocampal neurons of mice (Fig. 5C). Additionally, the expressions of ATG5, ATG12, and LAMP-1 in the hippocampus of APP/PS1 mice were lower than those in WT mice (Fig. 5D–H). Meanwhile, AST efficiently up-regulated the ATG5, ATG12, and LAMP-1 expressions in a dose-dependent manner, among which treated with 40 mg/kg AST was especially meaningful (Fig. 5D–H). The results showed that AST stimulated the formation of autophagosomes in hippocampal neurons of APP/PS1 mice.

### AST pretreatment improves the morphological alterations of HT22 cells damaged by Aβ25-35

**Fig. 6:**
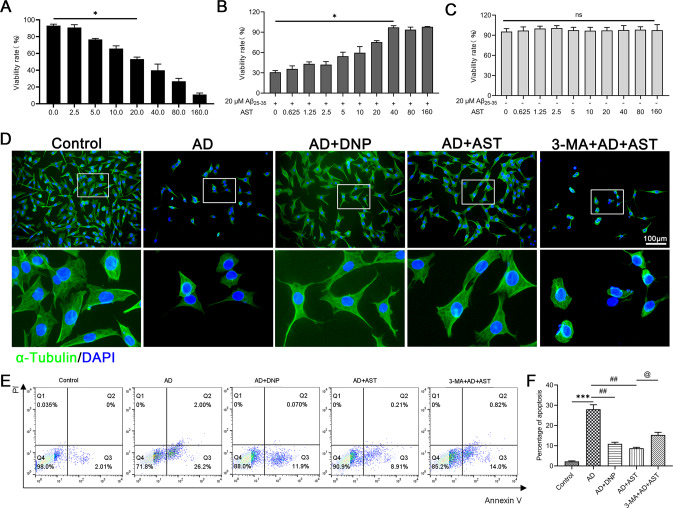
Effects of AST on the proliferation and apoptosis of Aβ25-35-damaged HT22 cells. **A** Cell viability was detected in HT22 cells with different concentrations of Aβ25-35 (0–160 μM Aβ25-35, 24 h) by CCK8 assay. **B** Cell viability was detected in Aβ25-35-injured HT22 cells (20 μM Aβ25-35, 24 h) with different concentrations of AST (4 h before Aβ25-35 treatment) by CCK8 assay. **C** Cell viability was detected in HT22 cells with different concentrations of AST (0–160 μM AST, 24 h) by CCK8 assay. Data were presented as mean ± SD, *p < 0.05 vs 0 μM**. D** Effect of AST on the morphological alteration of HT22 cells was observed by immunofluorescent staining. **E**, **F** Flow cytometry analysis indicated the anti-apoptotic effect of AST on the apoptosis of HT22 cells. Data were presented as mean ± SD, *n* = 3/group. ***p < 0.001 vs control group, ^##^*p* < 0.01 vs AD group, ^@^*p* < 0.05 vs AST + AD group.

We further investigated whether AST had a protective effect on Aβ25-35-injured HT22 cells. To select the optimum concentration of Aβ25-35 and AST in further experiments, we examined the relative cell survival rate induced by Aβ25-35 and AST with various concentrations. As shown in Fig. 6A, cell counting kit-8 (CCK8) assay results showed 20 μM Aβ25-35 significantly decreased the survival rate of HT22 cells (Fig. 6A). However, pretreatment with 40 μM AST, the survival rate of Aβ25-35-injured HT22 cells was (97.17 ± 2.50) % (Fig. 6B). Also, no significant changes were observed in AST pretreated cells, indicating these concentrations of AST were safe for HT22 cells (Fig. 6C). And thus 20 μM Aβ25-35 and 40 μM AST were chosen in following experiments.

To better estimate the effects of AST on Aβ25-35-injured HT22 cells, we used donepezil (DNP), one of the four drugs approved by the FDA for the treatment of AD, as a positive control drug in this study. Additionally, α-Tubulin, a marker of skeletal proteins, was used to observe the effect of AST on the morphology of HT22 cells injured by Aβ25-35. Compared with control group, Aβ25-35-injured HT22 cells were wrinkled and the intercellular gaps were significantly larger (Fig. 6D). In contrast, pretreatment with DNP and AST, the HT22 cells of Aβ25-35-injured were larger and triangular or polygonal (Fig. 6D). As a specific inhibitor of autophagy, 3-Methyladenine (3-MA) can inhibit the occurrence of autophagy. However, inconsistent with cells in the AST-pretreated group, the cells in the 3-MA group became smaller and these cells were significantly wrinkled (Fig. 6D). In addition, compared to the control group, the apoptosis of Aβ25-35-injured HT22 cells was increased (Fig. 6E, F). Under the pretreatment of DNP and AST, the apoptosis of Aβ25-35-injured HT22 cells was inhibited, while the apoptosis level of HT22 cells was increased when AD cell models were cotreated with AST and 3-MA (Fig. 6E, F). These data suggested that AST had protective effect on Aβ25-35-injured HT22 cells.

### AST pretreatment enhances the expression of proteins involved in the regulation of autophagic flux in HT22 cells

**Fig. 7:**
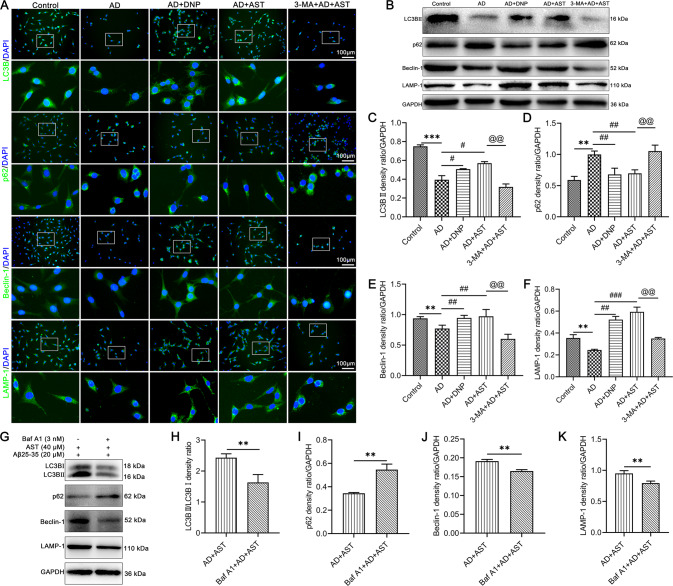
The expressions of LC3BII/LC3BI, p62, Beclin-1, and LAMP-1 in HT22 cells were detected by Immunofluorescent staining and WB detection. **A** Observation of LC3B, p62, Beclin-1, and LAMP-1 expression in HT22 cells by immunofluorescent staining, Scale bar = 100 μm. **B** Representative bands of LC3BII/LC3BI, p62, Beclin-1, and LAMP-1 in HT22 cells by WB detection. **C**–**F** Statistical analysis of the corresponding LC3BII/LC3BI, p62, Beclin-1, and LAMP-1 protein bands. Data were presented as mean ± SD, *n* = 3/group. **p < 0.01 vs control group, ^##^*p* < 0.01 and ^###^*p* < 0.001 vs AD group, ^@@^*p* < 0.01 and ^@@@^*p* < 0.001 vs. AST + AD group. **G** Representative bands of LC3BII/LC3BI, p62, Beclin-1, and LAMP-1 were detected by WB after the administration of Baf A1 in AD cell model. **H**–**K** Statistical analysis of the corresponding LC3BII/LC3BI, p62, Beclin-1, and LAMP-1 protein bands. Data were presented as mean ± SD, *n* = 3/group. **p < 0.01 vs AST + AD group.

To further confirm the character of AST on the regulation of autophagy or autophagic flux, we examined the expressions of LC3BII/LC3BI, Beclin-1, and LAMP-1 in Aβ25-35-damaged HT22 cells. Compared with the control group, the expressions of LC3BII, Beclin-1, and LAMP-1 were decreased and the expression of p62 was increased in HT22 cells damaged by Aβ25-35 (Fig. 7A–F). While administration of DNP and AST up-regulated the expressions of LC3BII, Beclin-1, and LAMP-1 and suppressed the expression of p62 in Aβ25-35-damaged HT22 cells (Fig. 7A–F). However, when AST-treated AD cell models were cotreated with autophagy inhibitors 3-MA or bafilomycin A1 (Baf A1), the ratio of LC3BII/LC3BI and the expressions of Beclin-1 and LAMP-1 were significantly reduced (Fig. 7B, C, E–H, J, K) while the expression of p62 was significantly increased (Fig. 7B D, G, I). These above results suggested AST activated autophagy and regulated the expression of autophagic flux-associated proteins.

### AST pretreatment reduces phosphorylation levels of PI3K/Akt-mTOR pathway-related proteins

**Fig. 8:**
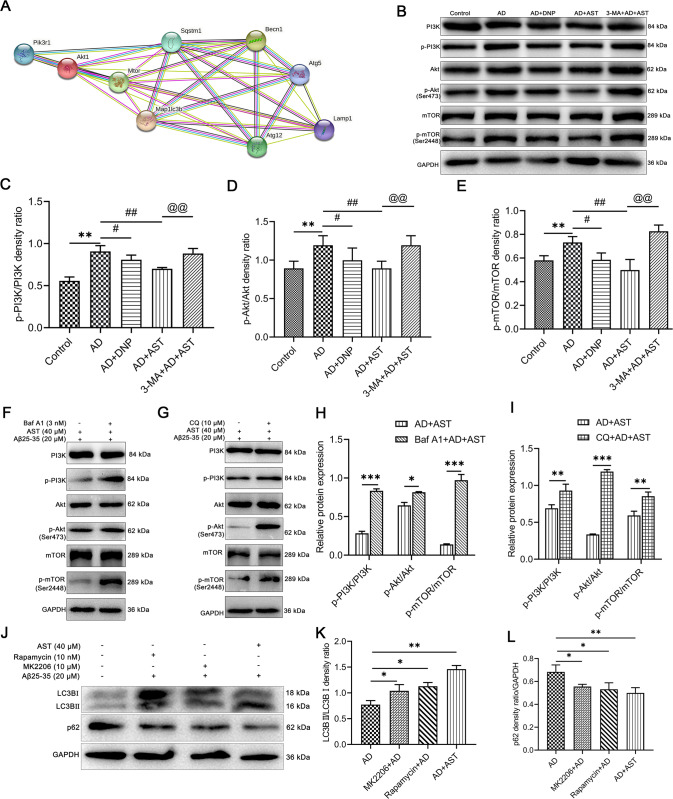
AST pretreatment decreased the phosphorylation levels of PI3K, Akt, and mTOR in the AD cells group. **A** Protein-protein interaction analysis of differently expressed autophagy-associated proteins using STRING database. **B** Representative bands of PI3K, p-PI3K, Akt, p-Akt, mTOR, and p-mTOR by WB detection in the HT22 cells. **C**–**E** Statistical analysis of p-PI3K/PI3K, p-Akt/Akt, and p-mTOR/mTOR in HT22 cells. Data were presented as mean ± SD, *n* = 3/group. **p < 0.01 vs control group, ^#^*p* < 0.05 and ^##^*p* < 0.01 vs AD group, ^@@^*p* < 0.01 vs AST + AD group. **F**, **G** The expression levels of PI3K, p-PI3K, Akt, p-Akt, mTOR, and p-mTOR were detected by WB after the addition of Baf A1 or CQ in AD cell model. **H**, **I** Statistical analysis of p-PI3K/PI3K, p-Akt/Akt, and p-mTOR/mTOR in HT22 cells of each group. Data were presented as mean ± SD, *n* = 3/group. *p < 0.05, ***p < 0.01 and ***p < 0.001 vs AST + AD group. **J** The expression levels of LC3BII/LC3BI and p62 were detected by WB after the addition of MK2206 or rapamycin in Aβ25-35-injured HT22 cells. **K**, **L** Statistical analysis of the corresponding LC3BII/LC3BI and p62 protein bands. Data were presented as mean ± SD, *n* = 3/group. *p < 0.05, **p < 0.01 vs AD group.

The PI3K/Akt-mTOR pathway exerts an imperative role in the central nervous system and is closely associated with AD pathology. Therefore, we first used STRING to analyze the protein interaction network and found that there were interactions between PI3K/Akt-mTOR pathway-related proteins and proteins regulated autophagic flux (Fig. 8A) (Source: 9 items (mouse)-STRING interaction network (string-db.org)). Then, we further evaluated the effect of AST on the PI3K/Akt-mTOR pathway. As shown in Fig. 8, the ratios of p-PI3K/PI3K, p-Akt/Akt, and p-mTOR/mTOR in Aβ25-35-injured HT22 cells were significantly increased to a varying degree when compared to those in the control group (Fig. 8B–E). Whereas the ratios of p-PI3K/PI3K, p-Akt/Akt, and p-mTOR/mTOR were distinctly reduced by administration of DNP and AST in HT22 cells damaged by Aβ25-35 (Fig. 8B–E). While when AST-treated AD cell models were cotreated with 3-MA, Baf A1, and chloroquine (CQ), the ratios of p-PI3K/PI3K, p-Akt/Akt, and p-mTOR/mTOR were significantly increased (Fig. 8B–I). In addition, compared with HT22 cells injured by Aβ25-35, the ratio of LC3BII/LC3BI was increased while the level of p62 decreased in Akt inhibitor MK2206, mTOR inhibitor rapamycin and AST treatment groups (Fig. 8J–L). The results further indicated that AST activated autophagy by inhibiting PI3K/Akt-mTOR pathway.

## Discussion

Our current study explored the role and potential mechanism of AST in autophagy and its implications on learning and cognitive function in AD mice. We demonstrated APP/PS1 mice displayed cognitive impairment and hippocampal neuronal damage, accompanied by autophagy dysfunction. However, AST significantly activated autophagy in hippocampal neurons of APP/PS1 mice, thereby attenuating hippocampal neuronal loss and cognitive impairment in APP/PS1 mice. Furthermore, the present study also indicated AST inhibited the PI3K/Akt-mTOR pathway in Aβ25-35-damaged HT22 cells. In summary, we revealed that AST may play a neuroprotective role in AD mice by prohibiting the PI3K/Akt-mTOR pathway to stimulate autophagy and regulate the expression of autophagic flux-related factors.

As a small molecule flavonoid, AST has been reported to further exert good hypnotic effects by prolonging the convulsion latency and reducing the convulsion rate in mice [16]. At the same time, studies have shown that AST transformed hippocampal microglia from M1 phenotype to M2 phenotype by targeting the IL-4R/JAK1/STAT6 pathway and the ubiquitination modification of STAT6, thereby alleviating perimenopausal depression-like behaviors and cognitive deficits in the mouse model of perimenopausal depression [17]. In addition, experimental evidence from Sun et al. demonstrates that AST can attenuate oxidative stress-induced necroptosis and thus play a protective role in spinal cord ischemia-reperfusion injury [18]. However, there are relatively few studies on the function of AST in neurodegenerative diseases such as AD in vivo and in vitro. In the preceding investigation of AD, AST has been reported to improve cognitive deficits and neuronal damage in AD mice by up-regulating estrogen receptor expression and inhibiting the activity of GSK-3β [19]. Consistently, in our study, we revealed APP/PS1 mice showed impaired cognitive function and SPs deposition, and these could be rescued by the administration of AST. In the pathological process of AD, Aβ is considered to play an important pathogenic role and its deposition in the brain is the initial stride in the pathogenesis of AD, resulting in consecutive neuronal loss and cognitive dysfunction [20]. Therefore, we also explored neuronal loss and apoptosis in vivo and in vitro. As expected, our findings implied that AST ameliorated the reduction and damage of neurons in the hippocampal region of APP/PS1 mice. In addition, in vitro studies have shown that AST had protective effect on Aβ25-35-induced injury in HT22 cells. DNP has been reported to attenuate AD pathology by protecting against neuronal damage [21]. In the present study, DNP was used as a positive agent for AST, and we found that like DNP, AST also reduced the morphological alteration and neuronal apoptosis of HT22 cells damaged by Aβ25-35. Accordingly, our study indicated that AST could ameliorate cognitive impairment, alleviate Aβ pathology in the brain, and decrease neuronal loss and apoptosis of AD progress.

Several evidences support that autophagy dysfunction plays an indispensable impact in AD pathology [22, 23]. Autophagy is a biological procedure in which eukaryotes utilize lysosomes to diminish organelles and toxic proteins and consists of a series of processes in which autophagosome structures are formed gradually. This dynamic process is called autophagic flux. In early AD, autophagy accelerates the clearance of misfolded proteins and promotes neuronal survival, and as AD progresses, the normal autophagic function of neurons is continuously destroyed and the autophagic flux process is blocked, and the ability to clear protein aggregates is declined [24]. Therefore, maintaining normal autophagy may be indispensable for attenuating the pathological process of AD. In our study, APP/PS1 mice displayed inhibited autophagic levels and autophagic flux was blocked in the hippocampus, while both could be rescued after the administration of AST. These results were consistent with the in vivo results, with the optimal effective concentration of 40 μM AST applied on Aβ25-35-exposed HT22 cells, the inhibited autophagy was activated and the levels of autophagic flux-related factors were up-regulated. We discovered a decrease in the LC3BII/LC3BI ratio and an increase of p62 expression in the hippocampus of APP/PS1 mice, while AST remarkably inverted this phenomenon. In vitro experiments, we also obtained similar results. Since autophagy is a dynamic process and is regulated by a series of key autophagy-related genes, such as Beclin-1, ATG5, ATG12, and LAMP-1. Beclin-1, a key protein in the initiation of autophagy, mediates the localization of other autophagy-related genes, and its elevated level indicates autophagic activation [25]. Herein, we confirmed the decreased expressions of Beclin-1 were reversed predominantly by AST in APP/PS1 mice as well as Aβ25-35-injured HT22 cells. Interestingly, we observed higher level of autophagy in the 40 mg/kg AST-treated group than that in the control group with a lower level of p62 and a higher level of Beclin-1. Our results were consistent with the study of Wang JG et al. that autophagy was activated after administration of thioperamide and p62 levels were higher in WT mice than in the thioperamide group [20]. Additionally, Long CM et al. found that N-linoleyltyrosine exerted neuroprotective effects on AD mice by activating autophagy, and Beclin-1 levels in WT mice were lower after N-linoleyltyrosine applied to APP/PS1 mice [26]. Therefore, we hypothesized that the inhibited autophagy in the hippocampus of APP/PS1 mice might be obviously activated after the administration of 40 mg/kg AST, and the autophagy level continued to increase to ensure the smooth progress of the subsequent process. As an indispensable gene of the ATG12-ATG5 coupling system, ATG12 exerts an imperative role in the extension of the autophagosome membrane and the formation of autophagosome [27, 28]. In our results, AST could up-regulate the protein expressions of ATG5 and ATG12 after being applied to APP/PS1 mice, which revealed that AST ensured the normal function of the intermediate link of autophagic flux. In addition, LAMP-1 is not only a marker for the binding of autophagosomes and lysosomes but also an important marker for the smoothness of autophagic flux [29]. In our in vitro and in vivo results, the protein of LAMP-1 was in an increasing trend after AST was applied on APP/PS1 mice and Aβ25-35-exposed HT22 cells, indicating that AST may promote the fusion of autophagosomes and lysosomes by enhancing LAMP-1 levels. Therefore, we hypothesized that AST may promote autophagy and autophagic flux in AD. Whereas AST exerted actions differently in different disease models. It is reported that in the model of bronchial fibrosis, AST can effectively alleviate bronchial fibrosis promoted by reactive oxygen species by inhibiting the formation of autophagy in airway epithelial cells exposed to oxidants [30]. Also, AST can enhance the anti-cancer effect by inducing apoptosis and autophagy [31]. In consequence, we used some autophagic inhibitors in Aβ25-35-exposed HT22 cells to better explain the effect of AST on AD autophagy. As a classical autophagy inhibitor, 3-MA plays an effective inhibitory role in autophagosome formation and development. At the same time, Baf A1 acts an indispensable role in suppressing the binding of autophagosomes and lysosomes. We observed the activation of autophagy and the degradation of autophagic lysosomes in AST-treated cells were blocked after adding 3-MA or Baf A1, which proved that AST could induce autophagy in Aβ25-35-injured HT22 cells. Above all, these results showed AST may exert a neuroprotective role in AD process by stimulating autophagy and autophagic flux.

As an intricate catabolic process, autophagy or autophagic flux is monitored by multiple signaling pathways, among which the PI3K/Akt-mTOR pathway is not only related to autophagosome formation but also intimately associated with the pathological mechanism of AD [32]. PI3K is an intracellular phosphatidylinositol kinase, which is regulated by several growth factors and mediates cell autophagy and apoptosis, proliferation and differentiation, and cell membrane vesicle transport [33]. When it is activated, PIP3 is produced at the plasma membrane and binds to Akt, prompting Akt translocation from the cytoplasm to the inside cell membrane for phosphorylation and activation [33]. The mTOR is a downstream target of the PI3K/Akt pathway and a promoter of autophagy or autophagic flux, which is mediated by a series of neurotrophic factors (BDNF, NGF, NT-3, etc.) and growth factors (EGF, FGF, PDGF, etc.) to negatively supervise autophagy or autophagic flux [34]. In the pathological process of AD, the PI3K/Akt-mTOR signaling is over-activated, leading to neuronal hyperactivity, transmitting wrong signals, and promoting SPs deposition [35, 36]. There is growing evidence that enhancing neuronal autophagy levels by inhibiting activation of the PI3K/Akt-mTOR pathway can promptly clear SPs and tau proteins in the AD brain [37, 38]. Meanwhile, previous studies have reported that scoparone promoted autophagy and attenuated the inflammatory response by blocking the PI3K/Akt-mTOR pathway [39]. Here, our results showed AST prohibited the activation of the PI3K/AKT-mTOR pathway in Aβ25-35-injured HT22 cells, while autophagy inhibitors 3-MA, Baf A1, and CQ reversed this change. In addition, we applied Akt inhibitors MK2206 and mTOR inhibitors rapamycin on HT22 cells under Aβ25-35-induced injury to verify again whether AST activated autophagy through this pathway. Similar to the effects of inhibitors MK2206 and rapamycin, the up-regulation of the LC3B-II/LC3B-I ratio and the down-regulation of p62 in the AST group indicated that autophagy was activated. These results supported AST up-regulated autophagy or autophagic flux by inhibiting the PI3K/AKT-mTOR pathway. However, in the current study, we did not clarify the key target factors of autophagy that ensure the neuroprotective effects of AST. Therefore, we need to explore further in the relevant study.

In conclusion, we demonstrated that AST attenuated pathological features and alleviated cognitive dysfunction in APP/PS1 mice, as well as protected Aβ25-35-injured HT22 cells via activation autophagy or autophagic flux mediated by the PI3K/Akt-mTOR pathway. Our data revealed the effect of AST in the treatment of AD and provided experimental evidence for initiating clinical trials of AST as a treatment of AD by regulating autophagy process.

## Materials and methods

### Materials, antibodies and reagents

AST (B21704) with a purity of 98% and DNP (S60449) were obtained from YuanYe Bio-Technology Co., Ltd (Shanghai, China). Antibodies against p-mTOR (5536 S), mTOR (2983 S), p-Akt (4060 S), and Akt (4691 S) were all purchased from Cell Signaling Technology, Inc (Danvers, MA). Antibodies against p-PI3K (ab191606), PI3K (ab182651), LC3B (ab51520), p62 (ab91526), Beclin-1 (ab217179), ATG5 (ab108327), ATG12 (ab155589), LAMP-1 (ab62562), NeuN (ab104224) and GAPDH (ab181602) were all purchased from Abcam Biotechnology (Cambridge, MA). Antibody against Aβ1-42 (GT622) was purchased from Gentex Biotechnology (Southern California, USA). Aβ25-35 (A4559) was purchased from AnaSpec, Inc (San Jose, CA), and 3-MA (A8353) was purchased from APExBIO, Inc (Houston, USA). Baf A1 (GC17597), MK2206 (GC16304), and rapamycin (GC15031) were purchased from GIpBio, Inc (Chicago, USA). CQ (HY-17589A) was purchased from MCE, Inc (New Jersey, USA). The apoptosis detection Kit (FXP023) was obtained from Beijing 4 A Biotech Co., Ltd (Beijing, China). Mouse Aβ level was measured using an ELISA kit purchased from Jiangsu Meimian Industrial Co., Ltd (Jiangsu, China), and mouse Aβ42 level was measured using an ELISA kit purchased from Wuhan Fine Biotech Co., Ltd (Wuhan, China).

### Animals and treatment

A total of 28 male APP_Swe_, PSEN1_dE9_ (APP/PS1) mice and 7 male C57BL/6 (WT) mice of 8 months were purchased from Guangdong Medical Laboratory Animal Center, China (Permit Number: SCXK GUANGDONG 2018-0002). The mice were housed under a temperature-and humidity-controlled condition with food and water *ad libitum*. After 7 days of adaptation to the environment, APP/PS1 mice were randomly divided into APP/PS1 group, 10 mg/kg AST (APP/PS1 + AST 10) group, 20 mg/kg AST (APP/PS1 + AST 20) group and 40 mg/kg AST (APP/PS1 + AST 40) group, and the C57BL/6 mice were served as control group. Then mice were intraperitoneally injected with AST once daily for one month and mice in the WT and APP/PS1 groups were intraperitoneally injected with 0.1% DMSO configured with an equal volume of saline. All mice experiments were approved by the Ethics Committee of Guangdong Pharmaceutical University and were in accordance with the National Institutes of Health Guide for the Care and Use of Animals.

### HT22 cells culture and pretreatment

HT22 cells were purchased from Fuheng Biotechnology Co., Ltd (Shanghai, China). Cells were cultured (Dulbecco’s modified eagle medium containing 10% fetal bovine serum) at 37 °C in 5% CO2. The effects of different concentrations of Aβ25-35 and AST on the viability of HT22 cells were respectively detected by CCK8 assay according to the protocol. HT22 cells were then divided into the following groups: control group: HT22 cells without any treatment; AD group: 20 μM Aβ25-35 was added into HT22 cells; AD + DNP group: 20 μM DNP was added into the HT22 cells for 2 h, and then added 20 μM Aβ25-35 [40]. AD + AST group: 40 μM AST was added into HT22 cells for 4 h and then added 20 μM Aβ25-35. 3-MA + AD + AST group: 2 mM 3-MA and 40 μM AST were added into HT22 cells for 4 h, and then added 20 μM Aβ25-35 [41]. Baf A1 + AD + AST group: 3 nM Baf A1 and 40 μM AST were added into HT22 cells for 4 h, and then added 20 μM Aβ25-35 [42]. CQ + AD + AST group: after HT22 cells were treated with 10 μM CQ and 40 μM AST for 6 h, 20 μM Aβ25-35 was added [43]. MK2206 + AD group: HT22 cells were treated with 10 μM MK2206 and 20 μM Aβ25-35 for 24 h [44]. Rapamycin + AD group: HT22 cells were treated with 10 nM rapamycin and 20 μM Aβ25-35 for 24 h [45].

### Bioinformatics analysis

The traditional Chinese medicine systems pharmacology database and analysis platform (TCMSP) database (https://old.tcmsp-e.com/tcmsp.php) was searched to attain the Chinese medicine chemical constituents of the therapeutic AD and then summarized into the Table 1. The tested gene proteins were input into the String Gene Interaction Network database (https://string-db.org/). The selected targets were searched in “Multiple proteins” and the PPI network map was drawn.

### SDA test

The SDA test was conducted to evaluate the learning and memory of rodents [46]. The experimental setup consisted of an avoidance reaction chamber (15 × 15 × 46 cm^3^) and parallel steel bars energized at the bottom. A circular insulation platform (diameter 4.5 cm, height 4.5 cm) was placed in the middle of the box. Each group of mice was put into the reaction box to adapt to the environment for 3 min and then connected to the power supply. The mice were stimulated by an electric current (36 V, 0.5 mA, 5 min) and then jumped back onto the platform to avoid injurious stimulation. Mice were trained consecutively for 3 days, and the experiment began 24 h later. Memory retention was evaluated by measuring the time when mice first stepped off the platform (step-down latency) and the number of times mice jumped off the platform within 5 min (number of errors).

### MWM test

The MWM test was performed to assess the spatial memory and learning and memory abilities of mice [47]. The MWM test was carried out in a round tank (120 cm in diameter and 60 cm in depth) containing opaque water maintained at 25 ± 2 °C and included positioning navigation test, space exploration test, and visible platform test. All animals underwent a 5-day hidden platform trial with regular training four times a day. Removing the platform on day 6, the animals were placed in the water from the same quadrant, and then the time of mice reaching the original platform and the swimming trajectory within 1 min were recorded. On days 7 and 8, the platform was raised to a position 1 cm above the water surface and placed in another quadrant, the number of mice crossing the target platform was recorded within 1 min.

### Hematoxylin-Eosin (HE) staining

The morphological alterations of the hippocampal region in each group of mice were observed by HE staining. After the completion of drug treatment and behavioral studies, mice were anesthetized with 1% pentobarbital sodium and perfused with 4% paraformaldehyde (PFA) via the aorta, and then brain tissue was quickly removed for post-fixation. After dewaxed and hydrated, washed with 0.1 M PBS buffer, the brain sections were placed into a hematoxylin staining solution for 5 min, rinsed under tap water after removal, and then differentiated with 1% hydrochloric acid alcohol for 3 s. Subsequently, the brain sections were stained in 1% ethanol eosin solution for 10 s and then placed in gradient ethanol and xylene, dehydrated, transparent, and sealed with neutral balsam. Finally, the brain sections were observed and photographed under the microscope, and the degree of hippocampal neuronal damage in each group of mice was according to the scoring criteria of Shi [48] and Pulsinelli [49].

### Nissl staining

Nissl staining was used to assess the damage to hippocampal neurons [50]. After dewaxing and dehydration, brain sections were stained in toluidine blue solution in a 56 °C thermostat for 40 min. The sections were then removed and placed in 75%, 80%, and 95% ethanol for 5 min, followed by 100% ethanol twice for 2 min and xylene twice for 5 min. The neutral resin was added dropwise above the tissues. Subsequently, these sections were observed under the microscope and photographed. The number of Nissl bodies in the hippocampus of each animal was calculated by Image J software.

### Fluorescent imaging of SPs in the brain of mice

After dewaxed and hydrated, the brain sections were repaired with 0.01 M citrate buffer (PH: 6.0) at high temperature for 25 min. After blocking the non-specific antibody binding site, the brain sections were reacted with mouse anti-Aβ1-42 (1: 300) overnight at 4 °C. After washing with 0.1 M PBST buffer repeatedly, the brain sections were incubated with goat anti-mouse IgG H&L (Alexa Fluor® 594) (1: 800) at 37 °C for 1 h. Then further washing with 0.1 M PBST buffer, the brain sections were incubated with 0.3% thioflavin S in 50% ethanol for 20 min and then washed in gradient ethanol solution and double distilled water for 5 min. Finally, brain sections were observed by fluorescence microscope and the number of SPs was counted by Image J software (NIH, Bethesda, MD, USA).

### Enzyme-linked immunosorbent assay (ELISA)

To evaluate the levels of Aβ and Aβ42 in the serum, the fresh blood of animals was first obtained and then serum was collected before the mice were sacrificed. The levels of Aβ and Aβ42 levels were evaluated by ELISA kits following the manufacturer’s instructions. Finally, the absorbance at 450 nm was measured using microplate reader, and the sample concentration was calculated from Curve Expert 1.4 software (Hyams DG, Starkville, MS, USA).

### Cell viability assay

The 6 × 10^3^ HT22 cells were seeded in 96-well plates for 24 h and then were treated with different concentrations of Aβ25-35 and AST. 10 μl of CCK8 reagent was added to per well and incubated at 37 °C for 2 h in the dark. Finally, the absorbance of the samples under 450 nm was measured with a Bio-Rad microplate reader. To assess the impact of AST on the apoptosis of Aβ25-35-injured HT22 cells, flow cytometry was carried out. For flow cytometric analysis, adherent HT22 cells treated with drugs were collected and stained with Annexin V/PI following the manufacturer’s instructions. And then the data were analyzed by flow cytometry software (Becton-Dickinson, CA, USA).

### Immunohistochemistry

Immunohistochemistry was conducted in brain sections and cultured HT22 cells. After dewaxed and hydrated, antigen retrieval, and blocking the non-specific antibody binding site, the brain sections were incubated with rabbit anti-LC3B (1: 300), rabbit anti-p62 (1: 200), rabbit anti-Beclin-1 (1: 100), rabbit anti-ATG5 (1: 200), rabbit anti-ATG12 (1: 100), rabbit anti-LAMP-1 (1: 200) and mouse anti-NeuN (1: 300) overnight at 4 °C. After washing 0.1 M PBST buffer repeatedly, the brain sections were reacted with goat anti-rabbit IgG H&L (Alexa Fluor 488) (1: 800) and goat anti-mouse IgG H&L (Alexa Fluor 594) (1: 800) at 37 °C for 1 h, respectively. Then further washing with 0.1 M PBST buffer, the anti-fluorescence quencher containing DAPI was added and incubated for 8 min in the dark at 37°C to stain nuclei. The cultured HT22 cells were fixed in 4% FPA and washed twice with pre-cooled 0.1 M PBS buffer. Then immunohistochemical protocol was performed using the same method as brain sections. Finally, the stained brain sections and cells were observed using a fluorescent microscope.

### Western blot (WB)

Mice were anesthetized with 1% pentobarbital sodium, sacrificed and the hippocampal tissues were quickly removed. The separated hippocampal tissues were lysed in pre-cooled RIPA lysis buffer and the supernatant was collected. Then the protein content in extracts was determined by the Pierce BCA Protein Assay, followed by electrophoresis, and membrane transfer. Then, the membranes were incubated with the following primary antibodies overnight at 4 °C: rabbit anti-LC3B (1: 3000), rabbit anti-p62 (1: 1000), rabbit anti-Beclin-1 (1: 500), rabbit anti-ATG5 (1: 2000), rabbit anti-ATG12 (1: 2000), rabbit anti-LAMP-1 (1: 1000), rabbit anti-PI3K (1: 1000), rabbit anti-p-PI3K (1: 1000), rabbit anti-Akt (1: 2000), rabbit anti-p-Akt (1: 2000), rabbit anti-mTOR (1: 1000), rabbit anti-p-mTOR (1: 1000), and rabbit anti-GAPDH (1: 5000). On the following day, the membranes were repeatedly washed with 0.1 M TBST buffer and then incubated with HRP-labeled goat anti-rabbit IgG (1: 8000) at room temperature for 2 h. The cultured HT22 cells were washed twice with 0.1 M PBS buffer and lysed in pre-cooled RIPA lysis buffer containing PMSF and subsequent experiments were performed using the same method as hippocampal tissues. The proteins were visualized by the automatic chemiluminescence imaging and analysis system and analyzed with Image J software.

### Statistical analysis

All experiments were repeated at least three times. All of the data were expressed as mean ± standard deviation (SD). And after passing the homogeneity test of variance, one-way ANOVA or two-way ANOVA was used to compare groups followed by Turkey or Student-Newman–Keuls test. For comparison between the two groups, statistical significance was determined by a two-tailed unpaired *t*-test. And *p* < 0.05 was considered statistically significant.

## Supplementary information

Authorship change agreement

Original Data File

## Data Availability

All datasets generated for this study are included in this article.
