# Supplementary material for: Neuroprotective effect of astragalin via activating PI3K/Akt-mTOR-mediated autophagy on APP/PS1 mice
Source: Cell Death Discov. 2023 Jan 21;9:15. doi: 10.1038/s41420-023-01324-1 (PMC9867706; doi:10.1038/s41420-023-01324-1)

**Neuroprotective effect of astragalin via activating PI3K/Akt-mTOR-mediated autophagy on APP/PS1 mice**

**Cui-Zhu Yang ^1^, Shu-Han Wang ^1^, Run-Heng Zhang ^1^, Jia-Hong Lin ^1^, Ying-Hong Tian ^2^, Ya-Qi Yang ^1^, Jing Liu ^1^, Yu-Xin Ma ^1,3^**

Fig. 4D


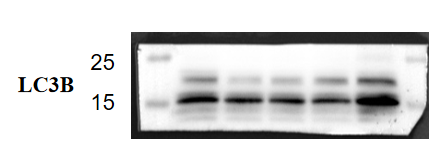


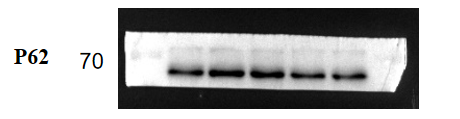


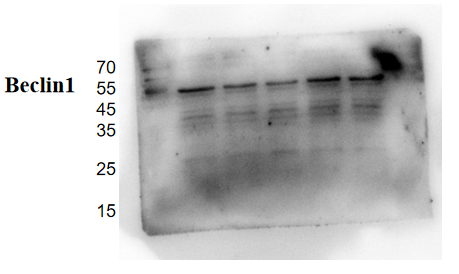


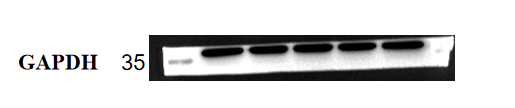


Fig. 5D


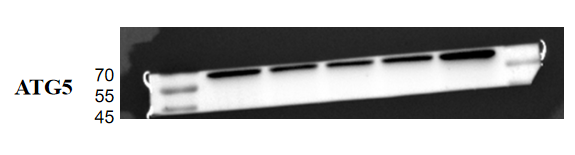


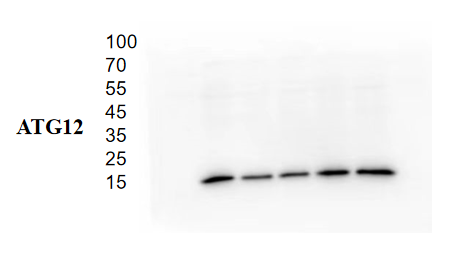


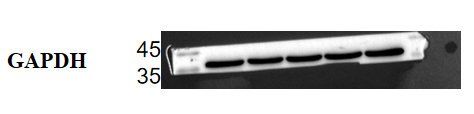


Fig. 5E


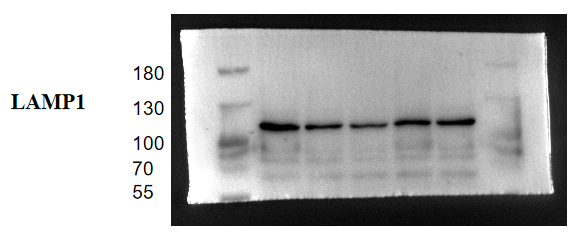


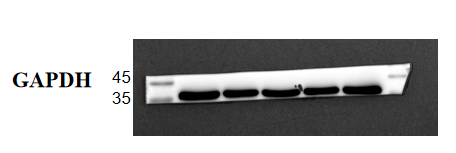


Fig. 7B


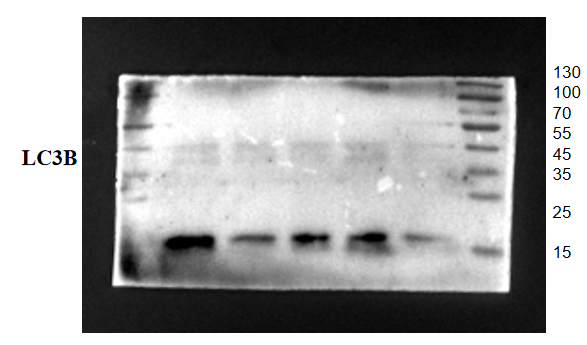


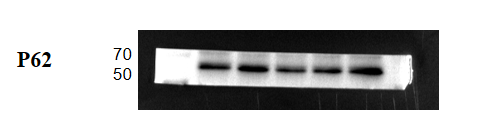


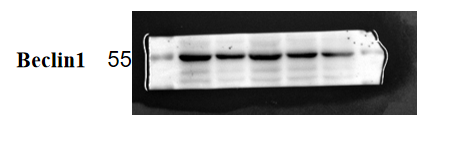


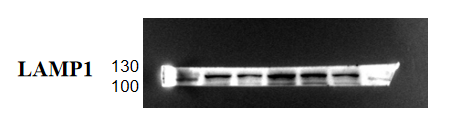


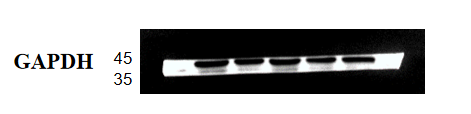


Fig. 7G


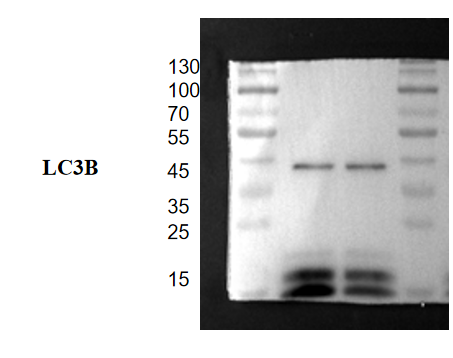


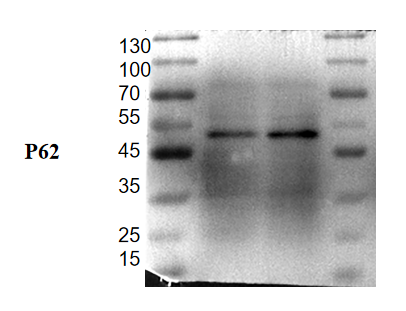


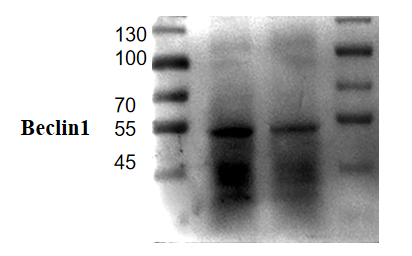


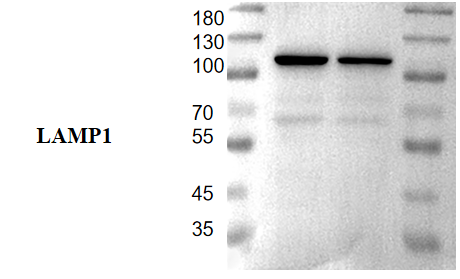

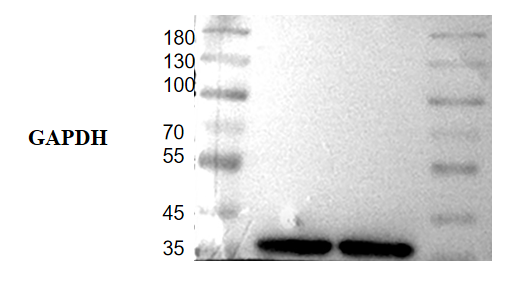


Fig. 8B


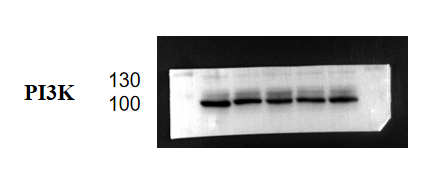


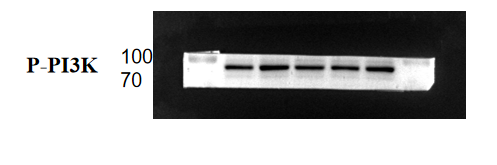


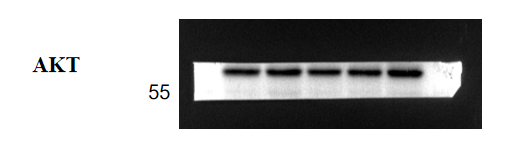


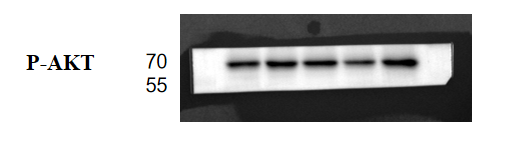


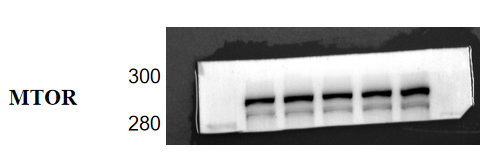


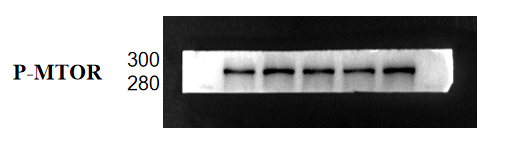


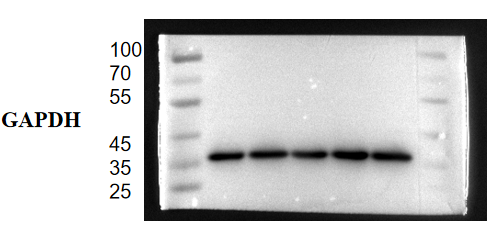


Fig. 8F


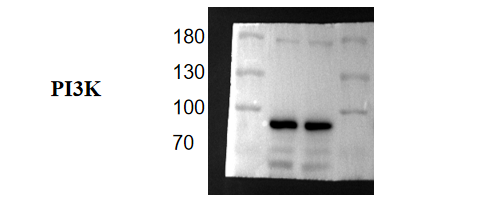


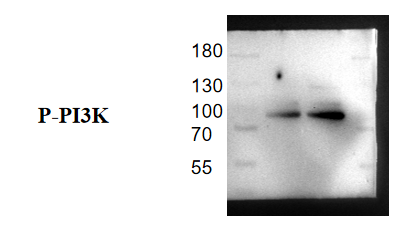


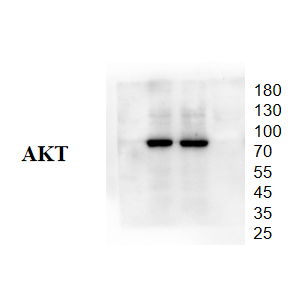


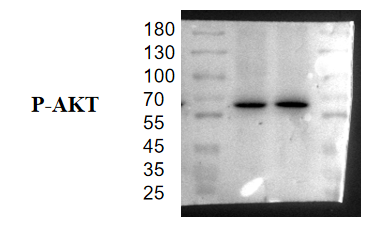

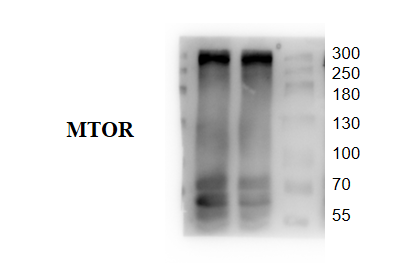

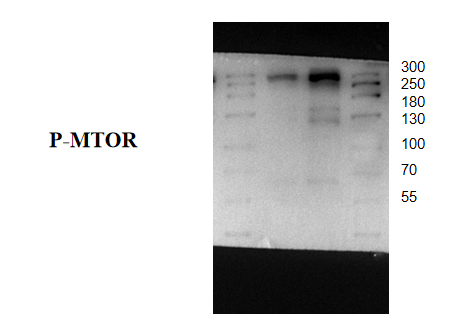


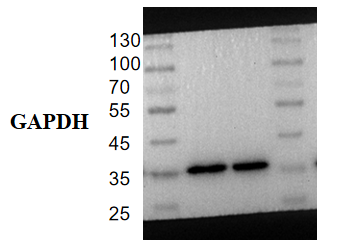


Fig. 8G


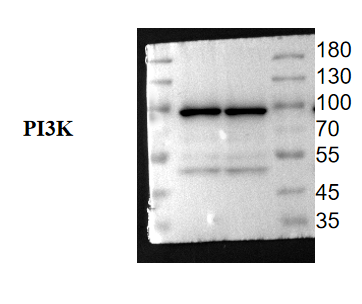


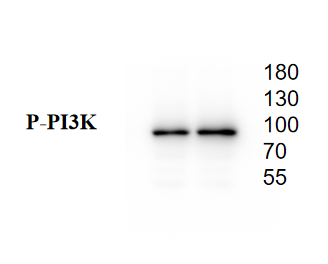


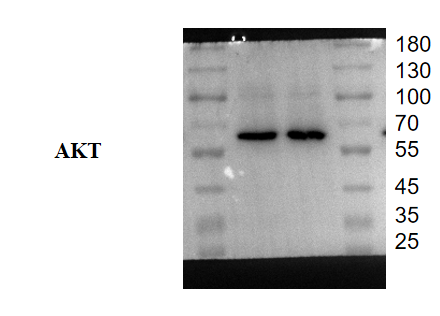


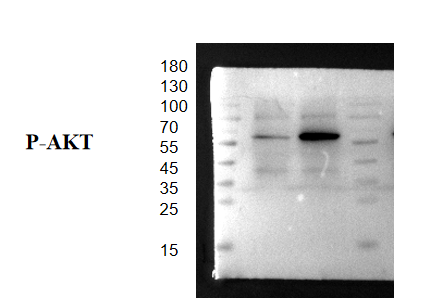

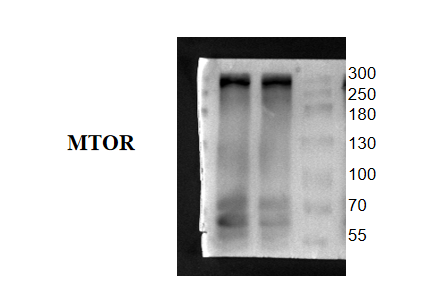

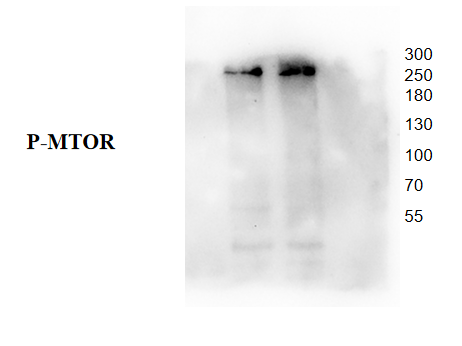

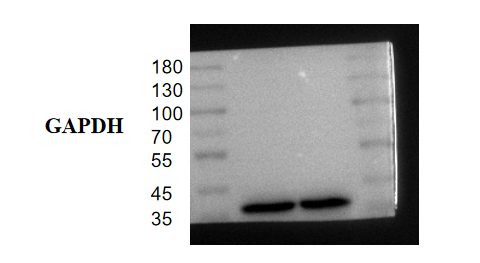


Fig. 8J


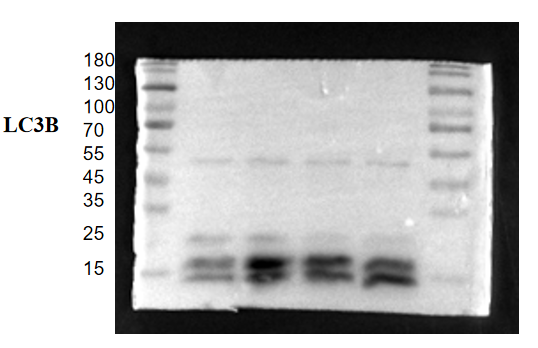

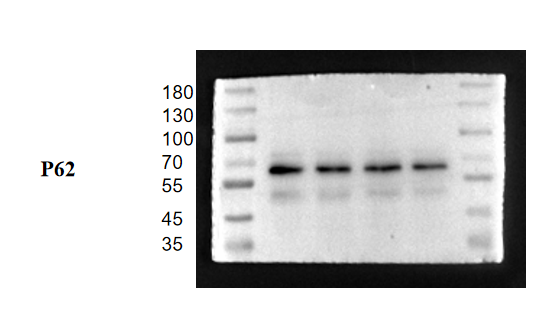

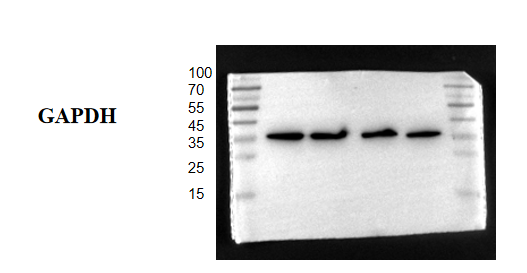

Supplement: Supplementary file 2 — Original Data File [file 41420_2023_1324_MOESM2_ESM.docx]
