# Supplementary material for: Neuroprotective effect of astragalin via activating PI3K/Akt-mTOR-mediated autophagy on APP/PS1 mice
Source: Cell Death Discov. 2023 Jan 21;9:15. doi: 10.1038/s41420-023-01324-1 (PMC9867706; doi:10.1038/s41420-023-01324-1)
Supplement: Supplementary file 1 — Authorship change agreement [file 41420_2023_1324_MOESM1_ESM.docx]

Dear Editor,

We would like to thank you for your friendly letter on our article (Manuscript Number: **CDDISCOVERY-22-4652R2**).

The order of authors for the **first** submission of our manuscript was according to Cui-Zhu Yang, Run-Heng Zhang, Shu-Han Wang, Ying-Hong Tian, Ya-Qi Yang, Jing Liu, and Yu-Xin Ma, with Yu-Xin Ma as the corresponding author. Because in the first submission, Yu-Xin Ma and Cui-Zhu Yang designed the study and wrote the paper. Cui-Zhu Yang, Run-Heng Zhang, and Shu-Han Wang performed the research. Ying-Hong Tian, Ya-Qi Yang, and Jing Liu provided professional and technical guidance for the paper.

During the **first revision** of the manuscript, the experimental additions and refinements were basically done by Shu-Han Wang, so we advanced Shu-Han Wang to the second author, and at the same time, Jia-Hong Lin assisted Shu-Han Wang to complete the additional experimental parts, so we placed Jia-Hong Lin as the fourth author.

When we revised the order of authors, we asked all authors and got their consent. At the same time, we reworked author contributions in the manuscript: MYX and YCZ designed the study and wrote the paper. YCZ, WSH, ZRH, and LJH conducted the research. TYH, YYQ, and LJ provided technical guidance. In addition, we sent a new email to all authors about the authorship order change and got confirmation emails from all authors. The screenshots of the specific email are below.

Thank you again for your professional and kind guidance on our manuscript, and let us all express our sincere gratitude to you. Have a great day.

Yours sincerely,

Yuxin Ma, Ph.D.

Guangdong Pharmaceutical University, Guangzhou, China
